# Physiological Effects of the Cool Vest Jacket on Recovery after a Repeated Shuttle Sprint Ability Test

**DOI:** 10.5114/jhk/218610

**Published:** 2026-04-02

**Authors:** Carlos Lorente-González, Jose Vicente Beltran-Garrido, Abraham Batalla-Gavaldà, Francisco Corbi

**Affiliations:** 1 National Institute of Physical Education of Catalonia, Faculty of Lleida, University of Lleida, Lleida, Spain.; 2 Physical Exercise and Performance Research Group, Department of Education Sciences, School of Humanities and Communication Sciences, CEU Cardenal Herrera University, CEU Universities, Castellón de la Plana, Spain.; 3 University School of Health and Sport (EUSES), Rovira i Virgili University, Amposta, Spain.; 4 Department of Education and Specific Didactics, Faculty of Humanities and Social Sciences, Jaume I University, Castellón de la Plana, Spain.; 5 Department of Clinical Sciences, Faculty of Medicine and Health Sciences, University of Barcelona, L’Hospitalet de Llobregat, Spain.

**Keywords:** physiological effects, cool vest, body temperature, soccer, metabolism

## Abstract

Soccer is an intermittent sport requiring rapid recovery from repeated high-intensity efforts, especially under heat stress conditions. Cooling vests have emerged as a practical strategy to enhance post-exercise heat dissipation, yet their physiological effects remain underexplored. This study aimed to assess the efficacy of a cooling vest following a repeated shuttle sprint ability (RSSA) test under hot conditions, focusing on skin temperature, blood lactate, and heart rate responses. Eleven recreational male soccer players completed two RSSA tests in a randomized crossover design, each followed by 15 min of passive recovery with or without a cooling vest. Skin temperature was measured at five anatomical sites, while blood lactate and the heart rate were recorded at baseline, pre-test, and at 0, 1, 3, 5, 10, and 15 min post-exercise. Compared with the control condition, the cooling vest intervention significantly reduced skin temperature at the 3^rd^ and the 5^th^ min post-exercise (3 min: d_z_ = −1.54, 95% CI [−2.53, −0.55], p < 0.001; 5 min: d_z_ = −0.90, 95% CI [−1.71, −0.08], p = 0.016). Transient between-condition differences were also observed for blood lactate at the 3^rd^ and the 5^th^ min (3 min: d_z_ = −1.00, 95% CI [−1.95, −0.006], p = 0.022; 5 min: d_z_ = −1.34, 95% CI [−2.36, −0.31], p = 0.003) and for the heart rate at 1 min post-exercise (d_z_ = −0.84, 95% CI [−1.59, −0.09], p = 0.013). No consistent differences were found at other time points. The protocol showed high between-day reliability (CV = 2.47%; ICC = 0.75), supporting the validity of the observed effects. In conclusion, post-exercise use of a cooling vest after repeated sprints in the heat accelerates early superficial thermal recovery, as evidenced by reductions in skin temperature during the first minutes of recovery. Transient and isolated differences were also observed for the heart rate and blood lactate concentration; however, these effects were not sustained across the full recovery time-course. From a practical perspective, cooling vests may be useful during short recovery windows in intermittent sports, while further research is needed to determine whether broader or longer-lasting physiological benefits can be achieved.

## Introduction

Soccer is characterized by substantial physical and tactical demands, requiring players to alternate high- and low-intensity phases throughout the 90 min of play. Across a match, players typically cover between 10 and 12 km, with approximately 60% of total playing time developing low-speed activities or walking (Ju et al., 2023). Despite occupying a small fraction of total time—often less than 10%—high-intensity actions such as sprints, jumps, and changes of direction frequently determine decisive moments in competition, being directly associated with goal- scoring opportunities, ball recovery, and successful tactical responses (Bortnik et al., 2024).

Repeated sprint ability (RSA) has been established as a fundamental determinant of performance in team sports (Bishop et al., 2011; Girard et al., 2011). RSA refers to the capacity to perform multiple short-duration sprints with incomplete recovery intervals, where anaerobic power, energy resynthesis efficiency, and neuromuscular recovery play a decisive role (Liu et al., 2024). In soccer, where success often depends on the repeated execution of explosive actions, RSA constitutes a critical component for sustaining competitive performance (Romero et al., 2025).

For these reasons, to evaluate this capacity, specific field-based protocols such as the repeated shuttle sprint ability (RSSA) test have been developed (Impellizzeri et al., 2008). Unlike linear sprint tests, the RSSA test incorporates changes of direction, thereby enhancing its ecological validity relative to match-play demands (Charron et al., 2020). Beyond physical performance outcomes, the RSSA test results also provide insight into physiological responses associated with accumulated fatigue, including lactate accumulation, heart rate dynamics, and thermoregulatory strain (Gharbi et al., 2014).

Fatigue elicited by repeated high-intensity efforts is mediated by both peripheral and central mechanisms. Metabolically, the accumulation of metabolites such as lactate and hydrogen ions disrupts muscle contraction and delays recovery processes (Fiorenza et al., 2019). From a cardiovascular perspective, the sustained elevation in the heart rate reflects the progressive increase in physiological loads as repeated efforts accumulate (Draghici and Taylor, 2016). In parallel, thermoregulatory strain—expressed as a progressive rise in body temperature—is linked to elevated perceived exertion (Hillen et al., 2023) and a consequent reduction in power output capacity. Additionally, variations in recovery duration have been shown to markedly influence internal and external loads during intermittent field-based efforts (de Dios-Álvarez et al., 2024), highlighting the sensitivity of recovery-dependent physiological responses.

Otherwise, in team sports the thermal environment represents a critical determinant of performance. Exposure to environmental heat not only compromises physical readiness but also impairs the capacity to sustain optimal performance (Modric et al., 2025). Exercise under hot conditions exacerbates physiological stress and accelerates the onset of fatigue. The elevation in core and skin temperature increases the energetic cost of thermoregulation, thereby diminishing the energy available for muscular work (Nybo et al., 2014). Simultaneously, heat-induced impairment in the central nervous system function can hinder cognitive performance and decision-making—both essential for success in soccer. The interaction between high-intensity intermittent effort and environmental heat thus creates an exceptionally demanding context that can markedly constrain athletic performance (Donnan et al., 2022).

Under these circumstances, the monitoring of physiological variables becomes essential to elucidate the body’s responses to repeated efforts under heat stress. Among the most widely used markers are blood lactate, the heart rate, and skin temperature (Takei et al., 2024). Post-RSA lactate levels typically range between 10 and 15 mmol·l⁻^1^—substantially higher than those observed in match-play where values seldom exceed 8 mmol·l⁻^1^ (Bishop et al., 2011)—and serve as a clear indicator of anaerobic performance and recovery capacity. The heart rate, conversely, rises sharply during the initial sprints and remains elevated throughout the test, underscoring the considerable cardiovascular cost of intermittent exercise (Jung et al., 2021). Meanwhile, skin temperature reflects both thermal strain and the efficiency of heat dissipation mechanisms, providing a relevant index for understanding recovery dynamics in hot environments (Priego Quesada et al., 2015).

Against this background, the identification of effective recovery strategies has become a central focus in sports science. Interventions such as cryotherapy, cold-water immersion, and ice-slurry ingestion (Fullagar et al., 2022), as well as the use of cooling garments, have demonstrated beneficial effects in lowering body temperature, alleviating thermoregulatory strain, and expediting the return to homeostasis (Nakamura et al., 2020). Recent evidence also shows that combined contrast heat-cold pressure therapy can accelerate post-exercise physiological recovery, reducing muscle soreness and improving recovery markers (Trybulski et al., 2024). Nevertheless, the practical implementation of these methods in competitive contexts is often limited by logistical constraints, the requirement for specialized equipment, or the time needed for their application.

Within this framework, cooling vests have emerged as a practical and promising alternative. Their portability, ease of use, and minimal interference with performance routines render them particularly suitable for applied sport settings (Fernández-Lázaro et al., 2023). Evidence indicates that cooling vests can decrease skin temperature, enhance subjective perceptions of recovery, and facilitate post-exercise physiological restoration (Otani et al., 2021), although the evidence base remains stronger for continuous rather than intermittent exercise. Moreover, most available research has focused on pre- or during-exercise applications, with limited evidence addressing their efficacy as a post-exercise recovery strategy following repeated sprint protocols under hot conditions (Seeley and Sherman, 2021).

Although cooling garments have been previously investigated, the available evidence is predominantly derived from studies applying cooling strategies before or during continuous exercise or over extended recovery periods. For instance, Otani et al. (2021) reported that a fan cooling jacket reduced thermal strain during post-exercise recovery under hot-humid conditions, while Seeley and Sherman (2021) showed that ice vests attenuated thermo-physiological strain following steady-state exercise in the heat. Furthermore, Chaen et al. (2019) demonstrated that wearing a cooling vest during half-time improved subsequent intermittent exercise performance; however, that study did not examine the temporal physiological recovery profile.

Importantly, none of these studies addressed the effectiveness of cooling vests as a post-exercise recovery intervention following repeated shuttle sprint protocols performed under field-based hot conditions, nor did they characterise the acute dynamics of skin temperature, blood lactate concentration and the heart rate within the short recovery windows (3–15 min) typical of in-play cooling breaks. Consequently, the applicability of cooling vests for rapid physiological recovery in team-sport contexts remains unclear.

Accordingly, the aim of the present study was to evaluate the efficacy of a cooling vest as a recovery strategy under hot environmental conditions, examining its effects on skin temperature, blood lactate concentration, and the heart rate.

## Methods

### Experimental Approach to the Problem

To evaluate the effects of a cooling vest as a recovery strategy following the RSSA test on skin temperature, blood lactate, and the heart rate, a within-subject crossover design was implemented. The RSSA test was performed twice, separated by 48 h, during which participants were randomly assigned to wear or not to wear the cooling vest during a 15-min recovery period after completing the test.

Skin temperature was measured using an Infrared Thermometer Iberia PCE-894 (PCE Ibérica, Tobarra, Albacete, Spain) in accordance with ISO 9886 guidelines (ISO 9886:2004, 2004). Blood lactate concentration was assessed with a Lactate Scout analyzer (Lactate Plus DP110, Diaglobal GmbH, Berlin, Germany) following the protocol described by Sánchez-Arjona (2008). Finally, the heart rate was monitored using Garmin™ chest straps (Garmin Ltd., Olathe, Kansas, USA) paired with UWB WIMU Pro™ devices (RealTrack Systems, Almería, Spain).

### Participants

The study sample consisted of 11 recreational male soccer players (age: 21.55 ± 1.04 years, body height: 174.18 ± 2.48 cm, body mass: 70.88 ± 3.48 kg, BMI: 23.35 ± 0.68 kg·m^−2^, VO_2peak_: 58.05 ± 1.62 ml·kg^−1^·min^−1^). The inclusion criteria were: i) participants did not engage in moderate- or high-intensity physical activity in the 48 previous hours, ii) participants had no personal or family history of cardiac injury and, iii) they did not suffer from any injury that could alter regular sports practice in the last 6 months prior to the study. None of the participants received any financial or in-kind reward for their participation in the study. They also signed an informed consent form, and a protocol was established for the delivery and explanation of the results. At the time of the study, none of the participants were taking any type of medication, nor were they following a specific dietary pattern or suffering from any respiratory or metabolic disorder.

### Procedures

**Aerobic Capacity Treadmill Test and Anthropometry:** Two weeks prior to the start of the study, a specialist in sports medicine conducted a treadmill test in the laboratory. The test began with a 2-min warm-up at 6 km·h^−1^, with progressive increments of 1 km·h⁻^1^ every minute. During the test, respiratory gas exchange was measured breath-by-breath using a metabolic cart (Quark CPET, Cosmed, Rome, Italy), calibrated prior to each test according to the manufacturer’s guidelines. VO_2__peak_ was determined as the highest 30-s average value attained during the test, with attainment criteria including a respiratory exchange ratio ≥1.10 and the heart rate within 10 beats·min⁻^1^ of the age-predicted maximum. In addition, body height was measured using a Seca® 220 stadiometer (Germany, accuracy of 0.1 cm), and body mass was assessed with a Seca® 700 column scale (Germany, accuracy of 0.05 kg). All tests and measurements were conducted in a controlled laboratory environment at a constant temperature of 22 ± 1.4°C and relative humidity of 32.3 ± 3.7%.

**Familiarization:** One week before the first study data registration, participants took part in a familiarization session during which the study protocol was explained and practice trials were performed; then, a written consent form was signed.

**Environmental Conditions:** Ambient temperature and relative humidity were recorded during both testing days using a portable weather station Kestrel® K4500 (Boothwyn, PA, USA).

**Test Scheduling:** The assessments were carried out across two testing sessions conducted at the same time of the day (10:00–12:00 a.m.), separated by 48 h of recovery. Upon arrival at the facility, baseline measurements of body height, body mass, skin temperature, the heart rate, and blood lactate were recorded. Participants then completed a self-paced warm-up consisting of dynamic mobility exercises, jumps, accelerations, and changes of direction, after which the same measurements were reassessed.

**RSSA:** In this study, RSSA was defined as “the ability to repeatedly perform maximal or near-maximal shuttle sprints with brief recovery periods”. To assess RSSA, we employed a protocol consisting of six 40-m shuttle sprints (20 m + 20 m with a 180° turn) interspersed with 20 s of passive recovery (Buchheit et al., 2010; Impellizzeri et al., 2008). Participants started behind the infrared photoelectric cells (Artek Pro®), sprinted 20 m to touch a cone, and returned to the starting line as quickly as possible. Upon crossing the photocell at the end of each sprint, players were instructed to decelerate immediately and return to the starting position, marked 50 cm before the first photocell (Gharbi et al., 2014). The photocell was positioned at ear height to prevent accidental triggering by arm or trunk movement. Each sprint was followed by 20 s of passive rest before the subsequent repetition. Following the standardized warm-up, players performed a single shuttle sprint to establish their criterion score (Impellizzeri et al., 2008), after which they rested for 5 min before commencing the test.

**Recovery with a Cooling Vest:** During the recovery period, participants remained at passive rest while wearing a cooling vest (Lorente-González et al., 2025). The vest (FlexiFreeze Ice Vest; Maranda Enterprises, LLC, Mequon, WI, USA) contained two reusable ice panels, each comprising 48 ice cubes positioned on the front and back, and secured with Velcro. The panels were frozen 24 h prior to use.

**Recovery without a Cooling Vest (Control Condition):** During the recovery period, participants remained at passive rest without wearing a cooling vest.

**Skin Temperature (Ts):** Skin temperature was recorded using an Infrared Thermometer Iberia PCE-894 (PCE Ibérica, Tobarra, Albacete, Spain), which has a resolution of 0.1°C and a measuring range of –50°C to +1850°C. Measurement precision within the temperature range of this study was ±0.5% of reading or ±2°C, according to the manufacturer. Measurements were conducted following the ISO 9886 protocol (ISO 9886:2004, 2004). Skin temperature was recorded at the following sites: the right scapula, the left chest, the left upper arm, the right anterior thigh, and the left calf. Data were collected at baseline, pre-test, and at 0, 1, 3, 5, 10, and 15 min post-test.

**Blood Lactate Measurements:** Blood samples were collected from the earlobe of each participant following the protocol described by Sánchez-Arjona et al. (2008). Blood lactate concentration was analyzed using a Lactate Scout photometer (Lactate Plus DP110, Diaglobal GmbH, Berlin, Germany). Measurements were taken at baseline, pre-test, and at 0, 1, 3, 5, 10, and 15 min post-test.

**Heart Rate Measurements:** The heart rate (HR) was recorded using Garmin™ chest sensors (Garmin Ltd., Olathe, Kansas, USA) with a sampling frequency of 4 Hz, paired with UWB WIMU Pro™ devices (RealTrack Systems, Almería, Spain) sampling at 100 Hz. This system had been previously validated for both HR (R^2^ = 0.96) and geospatial tracking (ICC = 0.98) (Molina-Carmona et al., 2018; Muñoz-López et al., 2017). Measurements were taken at baseline, pre-test, and at 0, 1, 3, 5, 10, and 15 min post-test.

### Statistical Analysis

The assumption of normality was verified using the Shapiro-Wilk test and exploring the Q-Q plots and histogram of residuals. The sphericity was checked using the Mauchly’s test and the Greenhouse-Geisser’s correction was applied when appropriate. Intra-day reliability of measurements was examined by the coefficient of variation (CV) and a two-way random intraclass correlation coefficient (ICC) with absolute agreement and 95% confidence intervals (CIs). Acceptable CV values were considered when CV ≤ 10% (Cormack et al., 2008; Turner et al., 2015). The ICC was interpreted as follows: ICC < 0.50 = poor, 0.5–0.74 = moderate, 0.75–0.9 = good and > 0.9 = excellent (Koo and Li, 2016).

To assess between-condition differences across the performance test scores, a two-way repeated measures ANOVA was used. The condition (i.e., intervention and control) and the time (baseline, pretest, 0 min, 1 min, 3 min, 5 min, 10 min and 15 min) were used as repeated measures factors. When a significant interaction effect was found, simple main effects were used to assess the between-condition differences, using the condition as the simple effect factor and the time as the moderator factor. To further quantify between-condition differences Cohen’s d_z_ (Lakens, 2013) effect sizes were calculated. Negative values indicate lower scores under the intervention versus the control condition. Effect sizes were interpreted as follows: < 0.2 = trivial; 0.2–0.6 = small; 0.6–1.2 = moderate; 1.2–2.0 = large; > 2.0 = very large (Hopkins et al., 2009). Researchers were blind to all subjects during analyses. The level of significance was set at α = 0.05 for all tests. All statistical analyses were performed in JASP for Mac (version 0.18.3; JASP Team (2024), University of Amsterdam, the Netherlands). Data are presented as mean ± SD.

## Results

### Environmental Conditions and Participants

No significant differences were observed in temperature, relative humidity, or wind between the two testing days. All tests were conducted under hot environmental conditions (31.8 ± 1.4°C, 39 ± 4.2% relative humidity, with a wind effect of 1.2 ± 0.4 m·s⁻^1^).

### Reliability Tests

The reliability data are presented in Table 1. Each measure had acceptable inter-day consistency with all CV values <5%, and moderate to good ICC’s ranging from 0.58 to 0.82. The between-sessions effect sizes were trivial to small with values ranging from 0.01 to 0.24, all *p* > 0.05.

**Table 1:** Test-retest reliability data for each assessment session.

Outcome	Day 1	Day 2	CV (95% CI)	ICC (95% CI)	ES (95% CI)
Sprint 1	7.32 ± 0.46	7.26 ± 0.36	1.91 (0.88, 2.94)	0.82 (0.47, 0.95)	0.24 (−0.37, 0.83)
Sprint 2	7.50 ± 0.44	7.47 ± 0.42	2.17 (1.25, 3.09)	0.80 (0.44, 0.94)	0.11 (−0.49, 0.70)
Sprint 3	7.60 ± 0.37	7.60 ± 0.41	1.96 (0.91, 3.00)	0.77 (0.37, 0.93)	0.01 (−0.58, 0.61)
Sprint 4	7.75 ± 0.43	7.65 ± 0.49	2.86 (1.69, 4.03)	0.69 (0.21, 0.90)	0.28 (−0.33, 0.88)
Sprint 5	7.85 ± 0.45	7.84 ± 0.57	3.25 (1.58, 4.91)	0.58 (0.04, 0.87)	0.02 (−0.58, 0.61)
Sprint 6	7.88 ± 0.55	7.85 ± 0.63	3.49 (1.79, 5.19)	0.65 (0.15, 0.89)	0.06 (−0.53, 0.65)
Mean	7.65 ± 0.42	7.61 ± 0.46	2.47 (1.54, 3.40)	0.75 (0.32, 0.92)	0.12 (−0.47, 0.71)

CV: coefficient of variation; CI: confidence intervals; ICC: intraclass correlation coefficient; CI: confidence intervals; ES: effect size

### Effectiveness of Conditions Groups

The summary of the between-condition differences is shown in Table 2, Figures 2 and 3.

**Table 2:** Descriptive statistics of the two conditions.

	Intervention (mean ± SD)			Control (mean ± SD)		
Time	Ts (Cº)	Blood lactate (mMol·L^−1^)	HR (bpm)	Ts (Cº)	Blood lactate (mMol·L^−1^)	Heart rate (bpm)
Baseline	32.40 ± 1.03	0.85 ± 0.27	54.09 ± 4.35	32.07 ± 0.80	0.87 ± 0.46	53.27 ± 5.95
Pre	34.02 ± 0.56	1.56 ± 0.54	110.64 ± 13.09	33.91 ± 0.55	1.60 ± 1.10	109.82 ± 13.30
0 min	35.64 ± 0.75	8.36 ± 2.31	185.18 ± 2.89	35.37 ± 1.05	8.70 ± 1.99	185.45 ± 4.44
1 min	35.37 ± 0.97	8.75 ± 2.12	145.91 ± 10.64*	35.70 ± 0.79	9.94 ± 1.64	152.18 ± 9.36
3 min	33.90 ± 0.60*	6.04 ± 1.84	122.91 ± 4.61	34.98 ± 0.57	7.53 ± 2.08	126.55 ± 5.24
5 min	33.60 ± 0.55*	3.50 ± 1.08*	111.36 ± 7.32	34.22 ± 0.32	5.48 ± 1.64	113.55 ± 6.70
10 min	33.45 ± 0.60	2.36 ± 0.64*	101.27 ± 4.94	33.72 ± 0.41	3.22 ± 0.78	102.00 ± 4.58
15 min	32.69 ± 0.59	--	90.18 ± 5.64	33.16 ± 0.57	--	92.45 ± 6.02

Data are presented as mean ± SD. Intervention: intervention condition wearing the cool vest during the recovery period, Control: control condition not wearing the cool vest during the recovery period; Ts: temperature of the skin; HR: heart rate. *: p < 0.05 statistically significant differences from the control condition

**Figure 1:**
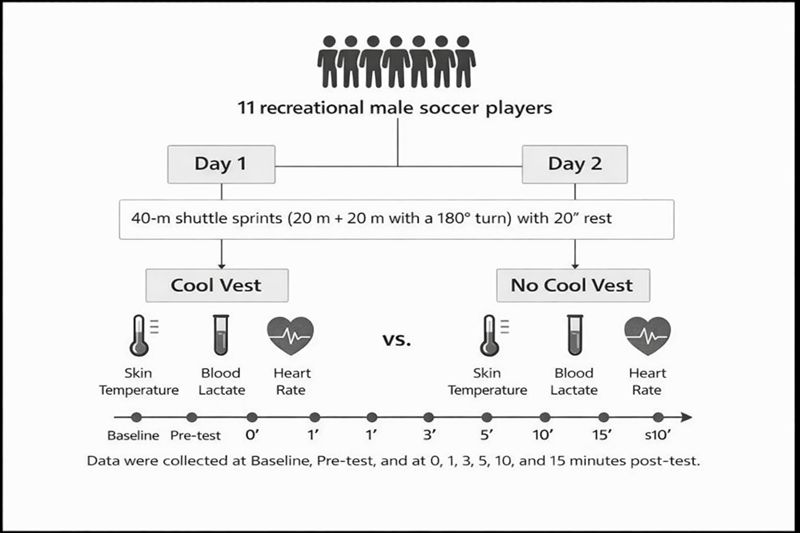
Schematic representation of the experimental design.

**Figure 2:**
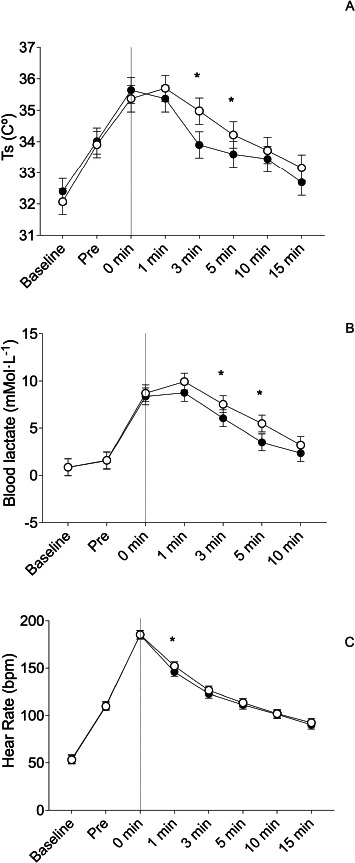
Between groups differences for A) skin temperature (Ts), B) blood lactate, and C) the heart rate. Black dots indicate the intervention group and white dots the control group. * p ≤ 0.050; the grey dashed line shows the end of the exercise protocol and the start of the recovery period

**Figure 3:**
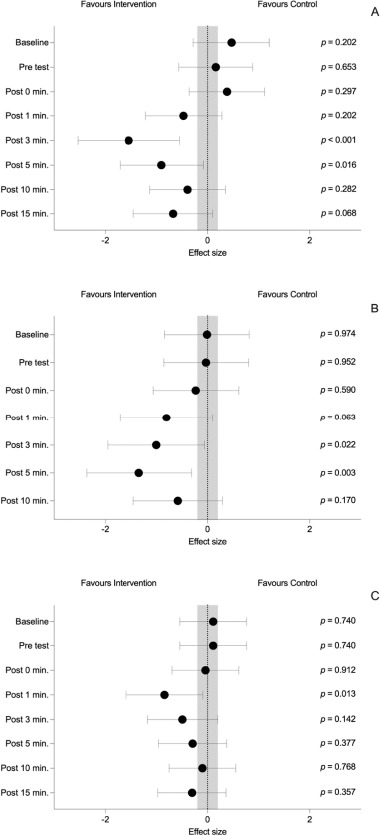
Between groups effect sizes and p-values for A) skin temperature, B) blood lactate, and C) the heart rate. Negative values indicate a favorable effect on the intervention group while positive values indicate a favorable effect on the control group. The grey dashed vertical lines delimit the lower (−0.20) and the upper limit (0.20) of a trivial effect size

Between-condition differences in Ts showed non-statistically significant differences at all time points (all *p* > 0.05) except for the third and the fifth minute of the recovery period, with lower values of temperature for the intervention condition (Post 3 min: d_z_ = −1.54 95% CI [−2.53, −0.55], *p* < 0.001; Post 5 min: d_z_ = −0.90 95% CI [−1.71, −0.08], *p* = 0.016) (Figure 1A and Figure 2A).

Between-condition differences in blood lactate concentration showed non-statistically significant differences at all time points (all *p* > 0.05) except for the third and the fifth minute of the recovery period, with lower values of blood lactate concentration for the intervention condition (Post 3 min: d_z_ = −1.00 95% CI [−0.06, −1.95], *p* = 0.022; Post 5 min: d_z_ = −1.34 95% CI [−2.36, −0.31], *p* = 0.003) (Figure 1B and Figure 2B).

Between-condition differences in the HR showed non-statistically significant differences at all time points (all *p* > 0.05) except for the first minute of the recovery period, with lower values of the HR for the intervention condition (d_z_ = −0.84 95% CI [−0.09, −1.59], *p* = 0.013) (Figure 1C and Figure 2C).

## Discussion

The main findings of this study indicate that wearing a cooling vest after an RSSA test under hot conditions accelerates superficial heat dissipation, as evidenced by lower skin temperature at the 3^rd^ and the 5^th^ min of recovery (moderate-to-large effects). In addition, early and isolated between-condition differences were observed in favor of the intervention for the heart rate (1 min) and blood lactate concentration (3 and 5 min). However, these effects were not sustained across the recovery period, suggesting that the physiological impact of post-exercise superficial cooling is most pronounced during the initial minutes and does not translate into prolonged metabolic or cardiovascular recovery.

These results may be related to the interaction between central and peripheral thermoregulation and post-exercise recovery processes. During high-intensity intermittent efforts, anaerobic metabolism rapidly increases heat production in active muscles (Jiang et al., 2023), accompanied by the accumulation of metabolites such as lactate and protons, contributing to increased muscle fatigue. Heat dissipation primarily depends on cutaneous evaporation and convection (Kenny and McGinn, 2017), processes that are compromised under high ambient temperature and moderate-to-high relative humidity, as in our study. This thermal load triggers cardiovascular adjustments, including an elevated heart rate and peripheral blood flow redistribution toward the skin (Périard et al., 2021) to facilitate heat dissipation and maintain homeostasis, albeit at the cost of accelerated muscular fatigue and reduced performance efficiency (Carroll et al., 2017). Cooling strategies, such as the use of a vest, enable heat removal from the skin surface via conduction and convection (Seeley and Sherman, 2021), reducing superficial temperature and indirectly modulating perceived exertion and thermal load.

Despite the reduction in skin temperature, the transient and early nature of the observed differences in blood lactate concentration and the heart rate suggests that superficial cooling primarily acts at a peripheral level (McMahon, 2024). Deep muscle temperature and internal metabolic processes may not be sufficiently affected by skin cooling, which could explain why the observed effects were limited to the early recovery phase and were not sustained across the full recovery period (McMahon and Kennedy, 2023). The intervention duration (15 minutes) and the limited cold penetration into deeper tissues likely constrained any systemic effects, which is consistent with previous studies showing that cold-water immersion or whole-body cryotherapy produce more pronounced and sustained reductions in blood lactate concentration and the heart rate (Vaile et al., 2008). This duration was chosen to reflect the recovery time typically available between match periods, including potential cooling breaks. Nonetheless, early reductions in skin temperature may be sufficient to improve thermal perception, decrease subjective thermal stress (Klous et al., 2020), and prepare athletes for subsequent efforts, particularly during short breaks such as halftime or intermittent rest periods.

From a cardiovascular perspective, although a significant between-condition difference in the heart rate was observed during the first minute of recovery, this effect was transient and not sustained across the full recovery period. Superficial cooling-induced vasoconstriction and peripheral blood flow redistribution may facilitate an earlier reduction in the heart rate (Koehn et al., 2020), supporting initial cardiovascular recovery without substantially altering mean heart rate values throughout the remainder of the recovery phase (Seeley et al., 2021). Superficial heat removal may also influence peripheral circulation and venous return, indirectly contributing to the restoration of homeostasis, although these mechanisms were not directly assessed in the present study.

Regarding blood lactate concentration, the observed pattern reflected the typical metabolic response following repeated high-intensity efforts, with immediate post-test values exceeding 8 mmol·l⁻^1^, confirming substantial anaerobic activation. Lactate clearance depends on factors such as muscle perfusion and oxidative capacity (Lucertini et al., 2017), which are unlikely to be markedly altered by superficial cooling. In this context, although significant between-condition differences in blood lactate concentration were observed at the 3^rd^ and the 5^th^ min of recovery, these effects were transient and not sustained across the full recovery period. The early reductions in blood lactate concentration observed with the cooling vest are consistent with previous findings reporting accelerated early lactate recovery following superficial cooling interventions (Huang et al., 2025), and may reflect indirect peripheral mechanisms, such as transient changes in blood flow distribution and reduced thermal strain (Lu et al., 2021) rather than meaningful alterations in muscle metabolic recovery. These findings align with prior studies describing modest and time-dependent effects of superficial cooling on metabolite clearance, whereas more invasive cooling strategies tend to induce larger and more sustained reductions in blood lactate concentration and the heart rate (Ihsan et al., 2016).

From a practical standpoint, these results indicate that the cooling vest represents an effective, safe, and easily implemented strategy during short breaks, reducing thermal load and improving comfort perception without interfering with tactical activity or force-generating capacity. In intermittent sports, where rapid recovery between high-intensity efforts is essential to maintain overall performance and prevent cumulative fatigue (Chaen et al., 2019), the vest provides a practical alternative to more complex methods such as cold-water immersion or whole-body cryotherapy, which require logistics, supervision, and may impair muscle function through local analgesic effects (Seeley and Sherman, 2021). Its simplicity allows coaches to integrate passive recovery without disrupting team dynamics, which is critical in sports with limited rest periods.

Despite these contributions, certain limitations should be noted. The study sample was small and homogeneous (11 young recreational male soccer players), limiting generalizability to elite athletes, female players, or other age groups. Only immediate physiological variables (skin temperature, lactate, and heart rate) were assessed, without evaluating effects on performance. Moreover, the intervention duration and initial tissue temperatures may have limited cold penetration to deeper structures, restricting effects on metabolic and cardiovascular recovery. Additionally, a sensitivity analysis conducted using G*Power (v3.1) indicated that, with a sample size of 11 participants, 8 repeated measurements, α = 0.05, and power = 0.80, the minimum detectable effect size was f = 0.30. This corresponds to a moderate effect size, suggesting that the study was adequately powered to detect moderate-to-large effects, but may have been underpowered to detect small effects. Consequently, subtle differences—particularly in blood lactate concentration and the heart rate—may have gone undetected. Moreover, although early between-condition differences were detected for the HR and blood lactate concentration at isolated time points, the study was not powered to robustly detect small and time-varying effects across the full recovery time-course. Thus, any non-significant contrasts at other time points—and the overall pattern—should be interpreted cautiously, particularly considering multiple comparisons and the small, homogeneous sample. Therefore, the absence of significant differences in some variables should be interpreted with caution, and future studies with larger samples are warranted.

Future research should explore combinations of superficial cooling with local cryotherapy or hydration-based interventions, assessing not only physiological variables but also functional performance outcomes. Expanding the sample to include women, elite athletes, and various age groups, as well as varying application times and vest designs to enhance cold penetration, could contribute to greater generalizability. Individual responses should also be considered, and subjective measures such as perceived exertion and thermal comfort should be included to provide a comprehensive evaluation. Additionally, the chronic effects of repeated vest use during training or competition, including potential physiological adaptations or cumulative benefits, warrant investigation.

## Conclusions

In conclusion, post-exercise use of a cooling vest after repeated sprints in the heat accelerates early superficial thermal recovery, as evidenced by moderate-to-large reductions in skin temperature at 3–5 min. Transient between-condition differences were also observed for the heart rate (1 min) and blood lactate concentration (3–5 min) in favor of the intervention; however, these effects were not sustained across the 15-min recovery period. From a practical perspective, cooling vests may be useful during short recovery windows (e.g., cooling breaks or half-time) to mitigate thermal strain, considering that broader or longer-lasting metabolic or cardiovascular benefits were not demonstrated with this protocol. Given the small and homogeneous sample, the exploratory nature of multiple time-point comparisons, and the minimum detectable effect size (f ≈ 0.30), these findings should be interpreted with caution and confirmed in larger and more diverse cohorts, ideally incorporating performance-related outcomes.
